# A Review of Perennial Ryegrass Endophytes and Their Potential Use in the Management of African Black Beetle in Perennial Grazing Systems in Australia

**DOI:** 10.3389/fpls.2017.00003

**Published:** 2017-01-19

**Authors:** Mijail Karpyn Esqueda, Alan L. Yen, Simone Rochfort, Kathryn M. Guthridge, Kevin S. Powell, Jacqueline Edwards, German C. Spangenberg

**Affiliations:** ^1^AgriBio, Department of Economic Development, Jobs, Transport and Resources, Centre for AgriBioscience, Agriculture Victoria, La Trobe UniversityMelbourne, VIC, Australia; ^2^Dairy Futures Co-operative Research CentreMelbourne, VIC, Australia; ^3^School of Applied Systems Biology, La Trobe UniversityMelbourne, VIC, Australia; ^4^Agriculture Victoria, Department of Economic Development, Jobs, Transport and ResourcesRutherglen, VIC, Australia

**Keywords:** endophyte, *Heteronychus arator*, pasture, pest management, control methods

## Abstract

The major insect pest of Australian cool temperate pastures is the root-feeding insect *Heteronychus arator* (African black beetle, ABB). Significant pasture damage can occur even at low ABB densities (11 individuals per square meter), and often re-sowing of the whole paddock is required. Mitigation of the effects of pasture pests, and in particular subterranean species such as the larval form of ABB, can be challenging. Early detection is limited by the ability to visualize above-ground symptoms, and chemical control of insects in soil is often ineffective. This review takes a look at the historical events that molded the pastoral landscape in Australia. The importation route, changes in land management and pasture composition by European settlers may have aided the establishment of ABB in Australia. Perennial ryegrass *Lolium perenne* is discussed as it is one of the most important perennial agricultural grasses and is widely-sown in moderate-to-high-rainfall temperate zones of the world. Endophytic fungi from the genus *Epichloë* form symbiotic relationships with cool season grasses such as *Lolium perenne* (perennial ryegrass). They have been studied extensively and are well documented for enhancing persistence in pasture via a suite of bioactive secondary metabolites produced by the fungal symbionts. Several well-characterized secondary metabolites are discussed. Some can have negative effects on cattle (e.g., ergovaline and lolitrems) while others have been shown to benefit the host plant through deterrence of insect pests from feeding and by insecticidal activity (e.g., peramine, lolines, ergopeptines). Various control methods for ABB are also discussed, with a focus on the potential role of asexual *Epichloë* endophytes.

## Introduction

Food production is a basic requirement for a sustainable society and the reason why a significant area of land has been dedicated to agricultural practices worldwide. Within these practices, part is devoted to animal production systems as grassland for grazing animals and hay production (Conant et al., 2001). Although annual grasses and food crops have been selected for their productivity since the beginning of agriculture, perennial grasses have only been studied in the last century (Wilkins, 1991). The domestication and expansion of grasses have been associated with the early stages of primitive agriculture in the Fertile Crescent of the Middle East about 10,000 years ago (Balfourier et al., 2000). This scenario suggests that ryegrasses were probably spread as weeds of cultivated crops by farmers during migratory events.

It has been predicted that the world population will increase by 50% between 2000 and 2050 to nine billion people (Kingston-Smith et al., 2013). Pastures play an important role in agriculture as production of meat and milk products increases to supply the growing human population (Lasley et al., 2009). This represents a challenge for agronomists, as they will have to achieve the right balance for sustainable production, one that does not compromise food quality or the environment (Tilman et al., 2002). Ruminants are vital for mankind as they provide high protein food products from plant material that is otherwise unsuitable for humans (Kingston-Smith et al., 2013). Finneran et al. (2012) highlighted the importance of determining the annual cost of feed in order to achieve a self-sustainable grassland system, therefore decreasing the purchase of concentrated feed and increasing profitability. Additionally, a better understanding of the nutritional requirements of ruminants has allowed for an improvement in the quality of forage crops grown on pasture land, which has translated into improved animal performance (Kingston-Smith et al., 2013).

However, growers face biotic (invertebrate pest, plant pathogens, and weeds) and abiotic (temperature, water, soil type, and nutrients, etc.) stressors that can severely reduce crop production; some of these biotic factors can be managed by physical (cultivation, mechanical weeding, etc.), biological (cultivar choice, crop rotation, predators, etc.) and chemical measures (pesticides, herbicides, Oerke, 2006). This review focuses on the control methods for the economically important insect pest, the African black beetle (*Heteronychus arator*) primarily focusing on the use of perennial ryegrass endophytes. Physical, biological, and chemical measures described by Oerke (2006) are explained here in terms of cultural control methods, natural predators and parasites, and chemical control. This review also includes a background on the beginnings of agriculture in Australia and a sequence of events that has led to the current pasture-based grazing systems for animal production.

## Early history of grazing in australia

The pastoral industry began in Australia at the time of European settlement. The first livestock to be introduced were bought from South Africa in 1787 and later transported to Australia (Clark, 1962). Due to the poor soil conditions and harsh grasses at the harbor side in Sydney the establishment of an agriculture enterprise was difficult (Younger, 1993). Initially, animals grazed on the cove of Port Jackson but were then moved to the head of the next cove after exhausting the limited feed in the area (Taylor, 1982). Even though early reports mentioned that most of the livestock imported survived after 6 months in Australia, all six cows and two bulls went missing after being left unattended (Younger, 1993). Food shortages and the lack of animals catalyzed the search for fertile soil leading to the settlement of the Rosehill area, 25 km west of Port Jackson, later that year (Hill, 2008).

In 1795, wild cattle were sighted in increasing numbers at the west bank of the Nepean River which were thought to be related to the cattle that escaped in the earlier years of the colony (Younger, 1993). It was not until 1803, however, that the government declared that the cattle could be maintained and numbers increased sustainably without relying on importation (Alexander and Williams, 1973).

Advances in food production were repeatedly affected by a series of droughts and insect plagues in 1810 forcing the colony to rely on imports from India, and in 1816 from Van Diemen's land (now Tasmania) (Stone and Garden, 1978). Food demand became a major concern as the number of convicts sent to Australia rapidly increased (Stone and Garden, 1978). Around 1820, meat production was the main focus for stockmen and other products, such as wool, were not as important at the time (Alexander and Williams, 1973). During the following years the agricultural industry experienced an expansion enabled by the convict system that provided cheap labor and inexpensive tracks of land (Stone and Garden, 1978). The earliest records of the dairy industry date back to the 1820s when dairy herds began to appear in the Illawarra district, New South Wales (Drane and Edwards, 1961).

The growth of the agricultural industry and the increase in investment allowed the establishment of the Australian Agricultural Company in 1824, which had large capital and land grants for expansion (Stone and Garden, 1978). The rapid expansion of the industry led to exploitation and grazing on unoccupied lands beyond the settlement boundaries despite the Governor's efforts (Billis et al., 1930; Alexander and Williams, 1973).

## Sequence of agricultural developments

Between 1830 and 1860, the Sydney-based colony expanded in all directions including southern Queensland, parts of the Riverina and across the Murray, Port Phillip, Adelaide, Swan River, and Albany in Western Australia (Pearson and Lennon, 2010). Agriculture in Australia suffered its ups and downs during this period. In the early 1840's an Australia-wide depression took place after a drop in prices of the main commodities at the time (wool and meat) due to a surplus of livestock (Alexander and Williams, 1973). During the gold rush in 1851, the pastoral industry was initially detrimentally affected but then bounced back due to the high demand for meat caused by the increase of migration into the country (Pearson and Lennon, 2010). Fencing and management of native and introduced pastures also came into practice during the acute labor shortage caused by the goldrush (Schofield, 1990).

With the rapid expansion of the cities, dairy herds were established to supply the locality with milk (Alexander and Williams, 1973). The location of dairy farms was determined by a range of factors including climate, topography but more importantly a nearby market (Drane and Edwards, 1961). To be able to provide milk twice a day to the nearby city, dairy farms required a high rainfall and a long growing season to favor introduced pastures such as perennial ryegrass (*Lolium perenne*), white clover (*Trifolium repens, T. pretense*) and prairie grass (*Ceratochloa unioloides*) (Alexander and Williams, 1973).

Until the 1880s, dairying remained a local industry as markets had to be near enough to enable the product to be transported before it spoiled (Drane and Edwards, 1961). In areas of high rainfall located away from the cities, cattle were kept for cheese and butter production (Alexander and Williams, 1973). Between 1880 and 1900, major technological advancements—including refrigeration for shipping, the Babcock system of estimating fat content of milk, and factory methods for manufacturing and preserving dairy products—allowed the rapid expansion of the dairy industry (Drane and Edwards, 1961).

From 1900 to the present time, the pastoral industry has gone through some challenging times: drought (1900), World War I (1910–1914), the Great Depression (1930–1940s), World War II (1939–1945), and recession (1990s) (Pearson and Lennon, 2010). During this period, however, scientific research focused on the selection and improvement of exotic and native grass cultivars to increase productivity and expand agriculture to areas which had not been exploited before (Parbery, 1967). In recent decades, the Australian industry has faced significant structural adjustment which has transformed the industry, driving productivity, and growth (Stott and Gourley, 2016). Milk production has intensified, with fewer farms and increased stocking rates, and there have been substantial increases in the use of bought-in feed and nitrogen (N) fertilizer to support increased milk production per cow and per hectare (CIE, 2011).

**Figure 1:**
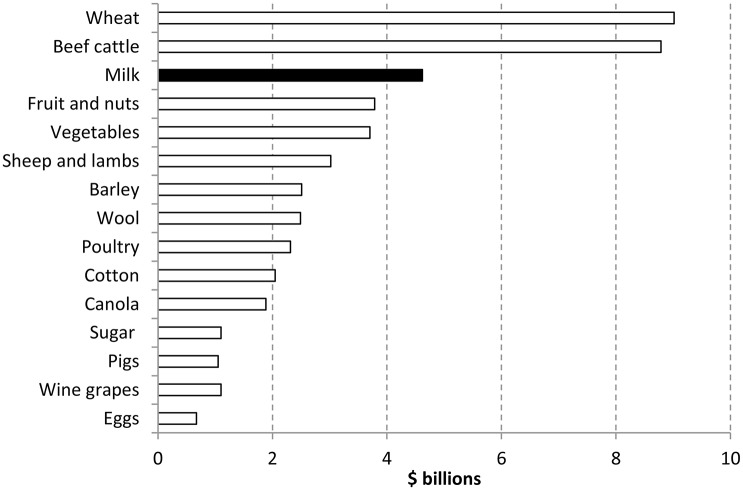
**Contribution of agriculture sectors in Australia 2013–2014 (ABARES, 2014)**.

Currently agriculture makes a significant contribution to the Australian economy both nationally and in regional areas. The value of farm production was almost $54 billion in 2013–14. Agriculture contributed around 2% of Australia's Gross Domestic Product with milk being the third highest commodity (See Figure 1) (ABARES, 2014).

Dairy farming relies on high quality permanent pastures for year-round grazing (Stott and Gourley, 2016). Pastures are typically dominated by perennial ryegrass (*Lolium* spp.) and varying proportions of legumes (e.g., clover, *Trifolium* spp., Chapman et al., 2008; Jacobs, 2014).

## Australian pastures

Prior to European settlement, extensive areas of vegetation were present either as grassland or as the understorey to *Eucalyptus* woodland in the south-eastern part of the continent (Groves et al., 2003). The typical plant composition of temperate grassland areas included perennial tussock, inter-tussock herbaceous flowering plants, with *Themeda, Poa*, and *Austrodanthonia* as the dominant grass genera (Lunt and Morgan, 2002). Vast areas of the temperate zone of south-eastern Australia had a long history of Aboriginal management before the first European settlers arrived on the continent (Gott, 2005).

Examples of land management by aboriginal people have been found in written reports of the earliest explorers:

“I found a considerable store of grass-seed, gum from the Mimosa, and other stores, carefully packed up in bags made from the skin of the kangaroo, and covered over with pieces of bark, so as to keep them properly dry. The weight of the bags containing the grass seed and gum was about 100 lbs; the seeds had been carefully dried after being collected from small grasses of the plains” (Coxen, 1866)

“Dry heaps of this grass, that had been pulled expressly for the purpose of gathering the seed, lay along our course for many miles” (Mitchell, 1848)

Additionally, Aboriginal people used fire to clear tracks and open hunting grounds (Rolls, 1999). It is no surprise that the temperate zone overlapped with the areas in which colonizers established and expanded their settlements (Pearson and Lennon, 2010). The extent of the temperate zone in the southern part of the continent incorporates Tasmania, most of Victoria, eastern NSW, areas of southern South Australia and the south-west of Western Australia (Dorrough et al., 2004). The European settlers exploited the fertile plains first, altering the native ecosystem to pasture and crops through agricultural practices (Lunt, 1991). Permanent changes in the vegetation composition such as pastures have occurred in response to the prolonged and intensive grazing (Groves et al., 2003). Large-scale conversion of grassland and grassy woodland to exotic pastures and crops took place on the fertile soils of south-eastern Australia as part of the colonization process (Fensham, 1998).

From the historical data we can appreciate how the improvement of pastures became an imperative in order to aid agricultural industries. The introduction of a number of plant species were promoted by the Acclimatisation societies, in conjunction with government botanists, during the late 1800s (Cook and Dias, 2006). Perennial pastures were first introduced into field trials in Victoria in 1860, as an initiative of the state government, with the purpose of evaluating the introduction and naturalization of pasture species (Cunningham et al., 1994). Perennial ryegrass was later reported as naturalized in Victoria before 1878 (Laffan and Ashton, 1964). Some studies date back to 1928 in Burnley, Victoria, when some pasture species were introduced and naturalized for agricultural purposes (Beilharz and Halloran, 1987). However, as pasture improvement moved further inland into drier regions the standard cultivars began to fail (Reed and Cocks, 1982).

In the 1950s, there was a large effort to develop perennial grass cultivars for the hotter, drier areas of south-eastern Australia. Grasses that could persist in a Mediterranean climate (average annual rainfall < 400 mm) were collected from Southern Europe and North Africa in order to provide new germplasm for breeding programs (Clark et al., 2016).

Cunningham et al. (1994) describes improvement programs of perennial ryegrass in Australia (1936–1995) from which different grass cultivars were developed and certified regionally (Victoria, New South Wales, Tasmania and South Australia) with the objective of improving their persistence, resistance to biotic and abiotic factors and quality.

More recently, in 1994, a joint effort by the Victorian Department of Agriculture, the U.S. Department of Agriculture and agencies in Morocco, Tunisia, and Italy collected widely in those three countries (Cunningham et al., 1997). Among the species collected were tall fescue, perennial ryegrass, phalaris and cocksfoot (Reed et al., 2016).

## Perennial ryegrass

Perennial ryegrass (*Lolium perenne*) is one of the most important perennial agricultural grasses world-wide. It is native to Europe, temperate Asia, and North Africa (Jensen et al., 2001) but it has been introduced in many countries including New Zealand, United States, and Australia for agricultural uses (Cunningham et al., 1994; Easton et al., 2001; Young et al., 2013), where it is widely-sown in moderate-to-high-rainfall temperate zones. Perennial ryegrass is an ideal forage grass due to its high digestibility, tolerance to grazing, and adequate seed production (Frame, 1989; Wilkins, 1991). Additionally, it is highly adaptive to different habitats and there is significant variation of traits in the wild populations providing room for genetic improvement (Wilkins, 1991).

There are three key factors that agronomists look to improve when breeding perennial ryegrass: dry matter yield, forage quality and persistence (temperature, drought, pests, disease, etc.) (Humphreys et al., 2006). As in most cool-season grasses, perennial ryegrass is an obligate outbreeder or self-incompatible plant that suffers from inbreeding depression (Cunningham et al., 1994). Self-incompatibility (SI) has been defined by Denettancourt (1977) as “the inability of a fertile hermaphrodite seed plant to produce zygotes after self-pollination.” Husband and Schemske (1996) defined inbreeding depression as “the reduction in fitness of progeny derived from inbreeding relative to those derived from outcrossing.” The SI mechanism in grasses hinders the production of inbred lines and hybrids in plant breeding, but also preserves heterozygosity in wild populations (Yang et al., 2008).

The obligate outbreeding nature of perennial ryegrass is why the initial advancements in breeding programs were performed by gene selection through sexual recombination (Humphreys et al., 2006). Continuous selection of full or half-sibling families allows for the improvement of pastures as desirable traits are assessed after each generation but progress is rather slow (Wilkins, 1991).

Another factor taken into account in perennial ryegrass breeding programs is the presence of asexual *Epichloë* endophytes because of the benefits of this symbiosis to the host plant (Funk and White, 1997). Elite cultivars obtained from breeding programs are combined with selected asexual *Epichloë* endophyte strains which can be incorporated into grass plants by inoculation (Funk and White, 1997).

## Fungal endophytes

*Epichloë* (syn. *Neotyphodium*) spp. have been described in early publications as endophytic fungi present in grasses such as perennial ryegrass and tall fescue (Sampson, 1933). They are an important group of filamentous fungi that infect cool season grasses, and consist of sexual (*Epichloë)* and asexual (*Neotyphodium)* species, previously classified as *Acremonium* sect. Albo-lanosa (Glenn et al., 1996). However, recent changes in fungal nomenclature rules have led to the renaming of the asexual (anamorphic) and sexual (teleomorphic) taxa into a single genus, designated *Epichloë* (Leuchtmann et al., 2014). The asexual growth of *Epichloë* endophytes in host grasses is characterized by seldom-branching hyphae in leaf sheaths which, in most cases, is aligned parallel to the leaf axis (Christensen et al., 2002). The endophyte is vertically transmitted through seeds by colonizing the developing flowers, ensuring their continuity from mother to daughter plant (Schardl, 1996). However, if the sexual stage of *Epichloë* species occurs, fungal stromata are formed on immature inflorescences “choking” affected flowering tillers and rendering them sterile (Moon et al., 2000).

The presence of asexual *Epichloë* endophytes in cool season grasses is not apparent and plants remain symptomless (Sampson, 1933; Wilson, 1993; Clay and Schardl, 2002; Iannone et al., 2011). However, infected plants can experience increased plant growth, reproduction and resistance to various biotic and abiotic stress factors (Clay and Schardl, 2002). For example, in symbiosis, *Epichloë* endophytes produce an array of secondary metabolites that benefit host plants through improved resistance against herbivores, pathogens, and drought (Siegel et al., 1987; Wilson, 1993; Zain, 2011). Christensen et al. (1993) demonstrated that alkaloid profiles are strain-specific producing the same chemical profile when the fungal symbionts are compared in the same plant. However, the chemical profile produced by an endophyte in symbiosis with a plant varies depending on the endophyte-plant combination (Panka et al., 2013). Evidence obtained from pot and field experiments have shown that endophyte-infected pastures perform better than endophyte-free swards under the above mentioned selective pressures (Prestidge and Gallagher, 1988b; Bacon, 1993; Crawford et al., 2010; Saikkonen et al., 2013).

Even though alkaloids produced by the endophytes can be beneficial for their host plant, they are also known to cause harm to vertebrate herbivores including livestock (Fletcher and Harvey, 1981; Crawford et al., 2010). Some groups of alkaloids have been identified as harmful, for example ergopeptine (ergovaline) and isoprenoid lolitrem (lolitrem B) which cause fescue toxicosis and ryegrass staggers, respectively (Smith et al., 1997). The effects of the different beneficial and harmful groups will be discussed in more detail in the section “Endophyte as a control method.” These bioactive properties have driven research on the alkaloids produced by endophytic fungi because of the services they provide to their host plant and agricultural systems (Table 1).

**Table 1:** **Chronology of some of the papers published on ryegrass endophytes**.

**Subject**	**Research description**	**Reference**
Animal health	Association of *Lolium* endophyte with ryegrass staggers.	Fletcher and Harvey, 1981
	Isolation of stagger-producing neurotoxins lolitrem A and B.	Gallagher et al., 1981
	Review on the effects and bioactivity of lolitrems, peramine, and paxilline. Lolitrem and paxilline have shown to be tremorgenic on vertebrates. In contrast peramine produces insect deterrence without affecting vertebrates.	Rowan, 1993
	Evaluation of the effects of penitrem, paxilline, and lolitrem B on sheep smooth muscle, show they cause low, mild, and persistence tremors, respectively.	Smith et al., 1997
	Review of mycotoxins important in ruminant feeding such aflatoxins, lolitrems, ergopeptine alkaloids, and others produced by fungi that are found in cattle feed.	D'Mello and MacDonald, 1997
	Evaluation of novel (AR37 and AR1) ryegrass endophytes showed improved persistence against insect pests without affecting cattle health.	Thom et al., 2013
Insect	Endophytes producing alkaloids responsible for ryegrass staggers in lambs (i.e., lolitrem B) were found to affect the growth rate of Argentine stem weevil (*Listronotus bonariensis*) larvae.	Prestidge and Gallagher, 1988a
	Endophyte infected plants exhibit increased insect resistance compared to uninfected conspecifics. Recommend that survey and selection of endophyte strains that do not affect cattle and benefit the host plant is necessary.	Clay, 1989
	Pot trials show that endophyte positive plants were significantly less damaged than endophyte free controls regardless of their alkaloid spectra.	Ball et al., 1994
	Bioassay based on mycotoxins found that only certain ergopeptine alkaloids deter adult African black beetle *in vitro*.	Ball et al., 1997b
	Absence of synergism between endophyte-infected perennial ryegrass and *Paenibacillus popilliae* against Japanese beetle (*Popillia japonica*).	Walston et al., 2001
	Pot trials found no effect of endophyte-infected ryegrass on redheaded (*Adoryphorus coulonii*) and blackheaded (*Acrossidius tasmaniae*) pasture cockchafers.	Watson, 2006
	Field trials examining the effects of selected endophyte strains (AR1 and AR37) and control against insect pests.	Popay and Thom, 2009
	Evidence of peramine and lolitrem B cascading up the food chain from aphids to ladybird increasing the duration of the pupal stage.	Fuchs et al., 2013
	Impact of selected endophytes (Wild-type, AR1 and AR37) and control against root aphids, African black beetle, Argentine stem weevil on field trials showed a decrease on insect pressure: Control > AR1 > Wild-type = AR37.	Thom et al., 2014
Plant performance	There is no effect of endophyte on photosynthesis and associated processes but there is evidence endophyte-infected plants are more tolerant of environmental abiotic stresses than uninfected grasses.	Bacon, 1993
	Leaf sheaths and leaf blades maintain similar peramine concentration, but decrease with leaf age. The seed from reproductive clones and younger sheaths and blades of leaves from vegetative tillers contained the highest concentrations, while the root, crown, and dead leaf tissue contained the lowest.	Ball et al., 1997a
	Grass-endophyte associations are based primarily on protection of the host from biotic and abiotic stresses.	Clay and Schardl, 2002
	Endophyte-infected plants promoted competitiveness, hindering weed invasion.	Saikkonen et al., 2013

## Pasture pests

Invertebrate activity can severely affect pastures by decreasing growth and establishment rate, impacting pasture composition favoring less palatable species and weeds, and enhancing damage caused by vertebrate grazers and predators by exposing areas to soil erosion (Bailey, 2007). However, some invertebrates which play a role in promoting pasture health (e.g., earthworms, termites, and ants) are regarded as soil engineers (Jouquet et al., 2006). Invertebrates that participate in biological, chemical and physical processes providing soil ecosystem services (e.g., recycling of nutrients, control of local microclimate, regulation of local hydrological processes, regulation of the abundance of undesirable organisms, and detoxification of noxious chemicals) as well interacting with other organisms in the substrate are recognized as beneficials (Altieri, 1999; Lavelle et al., 2006). It has been suggested that loss of biodiversity can prove costly for agroecosystems, as this directly affects basic regulation processes including soil fertility and pest control (Altieri, 1999).

In Australia, changes in land use from native pasture to intensive agriculture with exotic temperate pasture grass and legume species has led to addition of fertilizer and superphosphates to the soil to sustain such practices (King and Hutchinson, 1983). These landscape modifications have been associated with improved livestock production; however they also affect soil structure, water and nutrient cycling, as well as pasture productivity and palatability (Dorrough et al., 2004). Introduced pasture species influence their landscape by decreasing biodiversity of vegetation and invertebrate communities (King et al., 1985). In this large-scale intensive agriculture model, there is an increasing dependency on chemicals to manage pests which differs from the concept of sustainable agriculture (Tilman et al., 2002; Tscharntke et al., 2005).

European studies have found that improvement and management of pasture affects abundance and species richness of predators such as carabid beetles and spiders. Frequent use of the organo-phosphate pesticide chlorpyrifos was singled out as an important factor affecting predator richness (Rushton et al., 1989). In addition to the application of pesticides, grazing pressure has shown to have an impact on arthropod diversity of predator species (e.g., spiders) as well as affecting the abundance and diversity of pollinators such as bumblebee species (Tallowin et al., 2005).

In Australia there has been less research on the effect of agriculture on insect pollinators. Broad scale agriculture is thought to be associated with a low density of native bees, probably due to the absence of diverse nectar producing flowers, whereas the impact of pesticides on native bees is thought to play a more minor role as it is not well understood (Batley and Hogendoorn, 2009). Application of pesticides in perennial crops systems can be disruptive for beneficial insects, which is why refugia outside treated areas are essential (Landis et al., 2000). European studies suggest that mitigation of the negative effects of land management can be achieved by providing refugia adjacent to farmland to encourage the survival and reproduction of invertebrate predators (Macleod et al., 2004); changing grazing regimes to support beneficial species (Tallowin et al., 2005) and; reducing chemical sprays that impact on invertebrate predators (Rushton et al., 1989).

Although the relevance of European research to an Australian context is uncertain, Nash et al. (2008) found evidence to support transferability of some of this knowledge to Australian agricultural systems in regards to conservation of predatory invertebrates. An Australian research team, Tsitsilas et al. (2006), highlighted that grassy shelterbelts adjacent to pasture may influence the number of pest organisms. More importantly, these shelterbelts carried low numbers of pest species but higher numbers of predatory mites and spiders. Collins et al. (2002) found that although refugia within a crop field (in this study refered to as beetle banks) supported polyphagous predators, they failed to prevent aphid outbreaks; the presence of refugia did appear to have a significant impact on reducing the aphid population up to a distance of 83 m from the refuge. Collins et al. (2002) concluded that to prevent economic losses, optimal density of predators and spacing of refugia in fields must be determined.

Australia's major pest groups of grass pastures and turf have been well described by Bailey (2007), who provides detailed information about the pest's food source and some of the different control methods available (Table 2).

**Table 2:** **Major pest groups of grass pastures and turf in Australia, adapted from Bailey (2007)**.

**Pest**	**Common name**	**Scientific name**
Mites (Acari)	Cereal rust mite	*Abacarus hystrix*
	Blue oat mites	*Penthaleus* spp.
	Red legged earth mite	*Halotydeus destructor*
	Bryobia pasture mite	*Bryobia praetiosa*
	Balaustium mite	*Balaustium medicagoense*
Springtails (Collembola)	Lucerne flea	*Sminthurus viridis*
Snails and slugs (Mollusca)	Common garden snail	*Cantareus aspersa*
	Slugs	Eupulmonata
Caterpillars (Lepidoptera)	Black cutworm	*Agrotis ipsilon*
	Corbie	*Oncopera intricate*
	Winter corbies	*O. rufobrunnea*
	Underground grassgrubs	*O. fasciculate*
	Ghost moths	*Fraus simulans*
	Oxycanus grassgrub	*Oxycanus antipoda*
	Armyworms	*Leucania* spp.
	Pasture webworms	*Hednota* spp.
	Cotton webspinner	*Achyra affinitalis*
	Pasture tunnel moths	*Philobota* spp.
Crickets and Grasshoppers (Orthoptera)	Black field cricket	*Teleogryllus commodus*
	Mole crickets	*Gryllotalpa* spp.
	Wingless grasshoppers	Orthoptera: Acrididae
Beetles (Coleoptera)	African black beetle	*Heteronychus arator*
	Blackheaded pasture cockchafer	*Acrossidius tasmaniae*
	Redheaded pasture cockchafer	*Adoryphorus coulonii*
	Argentine stem weevil	*Listronotus bonariensis*
	White fringed weevil	*Naupactus leucoloma*

Historically, pasture pests have taken a toll on Australian agriculture from as early as 1810 when caterpillar plagues and drought severely affected pastures (Stone and Garden, 1978). Hoffmann et al. (2008) reviewed pest outbreak bulletins from the 1980–1984, 1985–1989, 1990–1994, and 2006–2007 from south-eastern Australia and reported that the relative incidence of lucerne flea, *Balaustium* mites, blue oat mites, redlegged earth mites, snails, and pasture cockchafers had increased during that period.

Scarabaeidae is one of the largest families of Coleoptera in Australia, comprising seven subfamilies and 3000 species (Allsopp, 1995). A number of these species are pasture beetles that share a similar lifestyle and behavior. For quite some time, all scarab larvae were commonly referred as “white grubs” because of their white/creamy color and curled shape during the larval stage (Cumpston, 1940). Members of the subfamilies Dynastinae, Rutelinae, and Melolonthinae are generally soil-dwelling, phytophagous, or phytosaprophagous, and in some cases the adults do not feed (Allsopp, 1995). However, there are still a number of soil-inhabiting pasture beetles whose larval forms have not yet been described (Berg et al., 2014). Pasture beetle larvae are predominantly a problem in grassland areas where they feed on humus and plant roots, decreasing plant persistence dramatically under stress conditions (e.g., grazing livestock, use of machinery, Blank and Olson, 1988; Berg et al., 2014). Some of the most cited crop and pasture pests in Australia from this family are summarized in Table 3. Most of these pasture beetles are endemic to Australia but that is not the case for the African black beetle, which, as its common name suggests, originates from Africa (Matthiessen and Ridsdill-Smith, 1991).

**Table 3:** **Scarabaeidae pests of crops and pasture in Australia**.

**Common name**	**Scientific name**	**Host plant**	**Reference**
African black beetle	*Heteronychus arator*	Blue gum, potatoes, tomatoes, grapevines, sugarcane, maize, kikuyu, phalaris, clover (*Trifolium* spp.) and *Paspalum* spp. *Lolium* spp.	Matthiessen and Ridsdill-Smith, 1991; Loch and Floyd, 2001; Bulinski et al., 2006; Bailey, 2007; Bell et al., 2011
Redheaded cockchafer	*Adoryphorus coulonii*	Subterranean clover, annual and perennial grasses	Bailey, 2007; Berg et al., 2014
Blackheaded cockchafer	*Acrossidius tasmaniae*	Annual grasses, legumes and cereals	Mcquillan, 1985; Bailey, 2007
Yellowheaded cockchafer	*Sericesthis harti*	Pasture and cereals	Bailey, 2007
Wheat root scarab	*S. consanguinea*	Pasture and cereals	Bailey, 2007
Black beetle	*Metanastes vulgivagus*	Pasture and cereals	Bailey, 2007
Black soil scarab	*Othnonius batesii*	Pasture and cereals	Bailey, 2007
Cockchafer	*Heteronyx obesus*	Pasture and cereals	Bailey, 2007

## African black beetle

African black beetle (*Heteronychus arator*) is a univoltine (1 year life cycle) soil-dwelling scarab beetle predominately found in grassland (Matthiessen, 1999; Bell et al., 2011). Temperature seems to affect, directly or indirectly, the presence and distribution of African black beetle, as its incidence has been associated with areas with mean annual surface temperatures greater than 12.8°C (Watson, 1979). African black beetle is recognized as an agricultural pest in Australia, New Zealand, and South Africa (Matthiessen and Learmonth, 1998). The earliest record of its introduction in Australia is a specimen collected in Newcastle, NSW, in 1920, but it is presumed to have become established prior to 1920 (Wright, 1958). The earliest record in the Australian Pest Plant Database dates back to 1930 (Plant Health Australia, 2001), similar to the first reports in New Zealand in 1937 (Todd, 1959). In Australia, African black beetle has been reported throughout the coastal region of New South Wales, widespread in pastures of south-western Western Australia, in coastal South Australia, parts of Queensland, and Victoria (Plant Health Australia, 2001). Most of its lifecycle occurs underground, but during the adult stage, they emerge to mate and on some occasions swarm (Ormerod and Janson, 1889; Matthiessen and Learmonth, 1998; Bulinski et al., 2006). It has been suggested that flights not only occur to vary habitat between life stages, but also to have a dispersive role; as seen in most dynastids (members of the subfamily Dynastinae), flights are an adaptation to the fluctuation between seasons (i.e., wet and dry, as well as, cold and hot, Watson, 1979).

African black beetle is a polyphagous species, reported to affect a number of different plants such as, blue gum (Loch and Floyd, 2001), potatoes (Matthiessen and Ridsdill-Smith, 1991), tomatoes, grapevines (Bulinski et al., 2006), maize (Drinkwater, 2003), sugarcane, clover (*Trifolium* spp.), and grass species including, kikuyu, phalaris, *Paspalum* spp., and *Lolium* spp. (Bailey, 2007; Bell et al., 2011). Larvae feed on the roots (Bell et al., 2011) while adults have been reported to cause severe damage to subterranean stems of seedlings, including young stems of potatoes and summer-sown crops (Matthiessen and Ridsdill-Smith, 1991; Erasmus and Berg, 2014).

One of the reasons African black beetle is a difficult pest to control is because of its potential to cause a high level of damage per individual (Bulinski and Matthiessen, 2002). It has been suggested African black beetle can cause significant damage to crops at densities exceeding 10 individuals per square meter (Bailey, 2007). Densities of over 100 larvae per square meter can cause direct damage to turf grasses, however secondary damage caused by foraging birds preying on the grubs can be observed even at lower densities (Ford et al., 2001).

**Figure 2:**
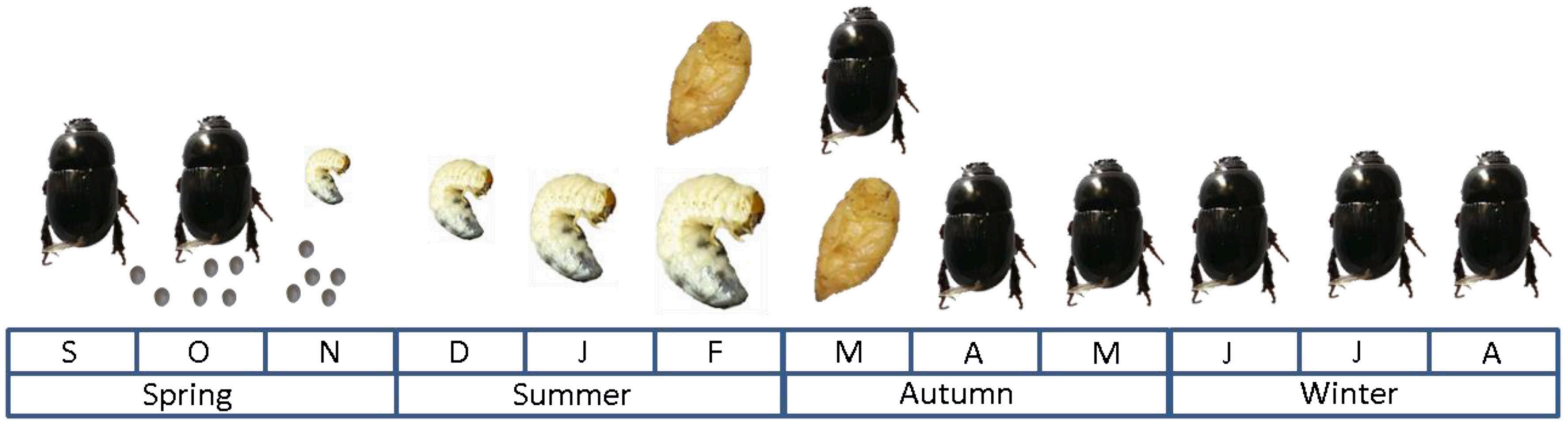
**African black beetle life cycle under Australian conditions as described by Matthiessen and Ridsdill-Smith (1991)**.

The African black beetle life cycle (Figure 2) starts in spring when the eggs are deposited, then the larvae go through three instars of development from around September-November to late summer when pupation takes place (Matthiessen and Ridsdill-Smith, 1991). Adults appear in numbers from March to September when mating and then oviposition occurs; adults die soon after reproduction (Matthiessen and Ridsdill-Smith, 1991). Flight dispersal mainly occurs during the autumn months by immature adults, while mature adults generally crawl during spring (Matthiessen and Learmonth, 1998). Autumn flights seem to be associated with the first significant rainfall after pupation, dusk surface temperatures (> 17°C) and favorable wind conditions (Watson, 1979). It has been suggested that flights during autumn and spring may play a crucial role in infestation of new pastures during outbreak years (Bell et al., 2011).

African black beetle was reported to be a serious crop pest in South Africa as early as 1889 (Ormerod and Janson, 1889). However, only two epidemic outbreaks (1946 and 1977) have been reported in South Africa, both in maize (Taylor, 1951; Drinkwater, 1979). African black beetle has been defined by King et al. (1981) as “a sporadic but serious pest of pastures and crops in northern areas of New Zealand's North Island.” The incidence of some outbreaks has been associated with warm conditions cause by a La Niña weather pattern (Eden et al., 2011). Warm spring temperatures greatly benefit African black beetle populations by allowing early oviposition followed by rapid egg and larval development, therefore increasing survival over summer (East et al., 1981). In Australia, African black beetle had been reported to reach plague levels in New South Wales as early as 1923 and subsequently in 1929–1933, 1936, 1940, 1944–1946, 1952–1954, 1957 (Wright, 1958). Additionally, a more recent study of pest outbreak reports from 1980 to 2006–2007 revealed that the relative incidence of pasture cockchafers, including African black beetle, increased during that period (Hoffmann et al., 2008).

## Control methods

### Cultural control methods

Cultural control methods refer to activities carried out to control one or more pests by changing the habitat conditions to promote biological control and/or decrease habitat quality for the pest (Horne and Page, 2008). As described in Bailey (2007), some cultural methods that could be used against African black beetle include: delaying sowing until November-December following the end of the beetles' life cycle; reducing potential habitat by removing grass and weeds from headlands; avoiding sowing in pasture areas that may contain adults; and establishing a physical barrier by cutting a deep furrow with a vertical side toward the crop. Some of the earliest remedies used to control African black beetle in South Africa included manure traps, sprinkling with salt, and application of lime into the soil, the latter being the only one reported as successful (Ormerod and Janson, 1889).

In Australia, some of the most productive agricultural land is naturally acidic (Scott et al., 2000). It has been suggested that addition of lime into soils with a naturally low pH may lead to local extinction of endemic acidophilic species, therefore its application must be treated with caution (Oliver et al., 2005). Furthermore, it is still unclear what effects lime applications might have on pasture cockchafers incidence and the host plant's ability to overcome feeding damage (Berg et al., 2014).

Several scarab species are considered significant pests of eucalypts (Frew et al., 2013). These include stem-feeders (Abbott, 1993) such as African black beetle (Paine et al., 2011) and common defoliators such as the Christmas beetles (*Anoplognathus* spp.) (Johns et al., 2004). It has been suggested that the use of fertilizer with nitrogen (N) in eucalypt plantations could be used as a management option as it has been found to either moderate or negate the effect of severe insect defoliation on growth (Pinkard et al., 2006a). This is because the leaf structure and texture of eucalypts may play a role on levels of herbivory (Sanson et al., 2001; Steinbauer, 2001). For example, Pinkard et al. (2006b) showed that leaf density or thickness of *Eucalyptus globulus* increased following N application in response to artificial defoliation. Therefore, it has been argued that nitrogen (N) application will not increase future herbivory problems (Pinkard et al., 2006a).

Nonetheless, irrigation and fertilization practices applied on eucalypt plantations have been positively correlated with an increase in scarab populations as these practices (mostly fertilization) also affect the understory (Frew et al., 2013). Therefore, even though fertilization with N prior to defoliation maintains stem growth and diameter at a similar rate to undefoliated unfertilized trees (Pinkard et al., 2007), it is concerning that the plantation understory of eucalypts and similar systems (e.g., orchards and oak woodlands) may serve as a potential niche for pasture beetles that will not only affect the plantation but also neighboring pastures and crops (Frew et al., 2013). Abbott (1993) highlighted that mat-forming grasses near plantations seem to favor African black beetle.

## Monoculture effects

Simplification of the environment on large expanses of land will cause an increase in the density of host plants, uniformity of crop population age structure and physical quality, and a decrease in biodiversity (Altieri et al., 1984). It has been suggested that pest problems in eucalypt plantations in south-western Australia may have been intensified by eucalypt monocultures (Loch and Floyd, 2001). Similarly, sown pastures are characterized by having lower diversity in vegetation and invertebrate communities than in naturally occurring pastures (King et al., 1985). In the case of pastures, it may be possible to break the lifecycle of the African black beetle through crop rotation; sowing non-host crops (e.g., brassicas, legumes, or chicory) in spring, thus causing a disruption of larval feeding as well as controlling grass weeds (Bell et al., 2011).

Even though evidence of monoculture effects has been found in agriculture (Altieri et al., 1984; Andow, 1991) this concept is not exempt from criticism (Emden and Williams, 1974; Goodman, 1975). Andow (1991) concludes that monocultures may influence pest abundance in different ways depending on the species (e.g., more in some, less in others) and that one hypothesis may not explain all insect-plant relationships. However, Root (1973) proposed two explanations for the monoculture effect and hypothesized that: (1) the level of complexity of the system is relative to the effectiveness of natural enemies to control herbivore populations (natural enemy hypothesis) and (2) specialized herbivores that can exploit the resources available in simple systems will reproduce in greater numbers than complex systems (resource concentration hypothesis). The facts that African black beetle is a polyphagous species (Bailey, 2007) and has the ability to disperse by flight (Matthiesse and Learmonth, 1998) should be taken into account when developing a management plan, as surrounding areas could represent a potential habitat for harboring this insect pest (Frew et al., 2013).

## Grazing

Grazing is a naturally occurring event that has an effect on the botanic diversity and structure of an area; for this reason, grazing is considered as a potential management tool in grassland conservation (Tallowin et al., 2005). However, it is essential to be able to foresee how grazing can affect vegetation in order to increase spatial heterogeneity rather than decrease it (Adler et al., 2001). Continuous selective grazing by livestock gradually deteriorates the quality and composition of grassland as it can cause the loss of the most palatable species of sward (Dorrough et al., 2004). Ultimately, grazing can alter landscapes by inhibiting regeneration of woody trees and native vegetation (Bennett et al., 1994).

Overbeck (2014) found that the influence of grazing on vegetation richness is relative to the productivity of the site, whereby low-productivity sites experienced a decrease in vegetation richness while high-productivity sites experienced an increase in vegetation richness. On the other hand, the level of grazing pressure has been found to affect beneficial invertebrates (e.g., bumblebees and spiders), as their abundance and species richness decreases under severe grazing regimes (Luff and Rushton, 1989; Tallowin et al., 2005).

It has been argued that plant and structural diversity in agricultural landscapes positively affects the abundance and diversity of natural predators of invertebrates thus offering improved biological control (Fiedler et al., 2008; Woltz et al., 2012). However, lack of knowledge on the biology and ecology of such predators represents a limiting factor when incorporating them into a management program (Horne and Page, 2008).

Some recommendations on grazing management for pasture beetle prevention include reducing cattle numbers in the affected paddocks early in the year when damage is at its peak (Blank and Olson, 1988; Berg et al., 2014) and reducing ground cover for egg-laying in early spring by heavy grazing and/or keeping pasture short (e.g., cut for silage, Douglas, 1972).

## Natural predators and parasites

The presence of natural predators affects pest-host plant relationships by hindering pests, causing them to utilize unsuitable areas that are less productive, or even cease feeding/reproduction completely; as a result, the outbreak phase can be delayed by controlling population numbers when they are below plague levels (Riechert, 1999). It has been suggested that pest regulation by natural predators plays a key role in the prevention of pest outbreaks in sustainable agricultural systems (Kromp, 1999). East et al., 1981 argues that predators of scarab pests are frequently insignificant in improved grassland. However, removal of overgrown grass has been shown to increase larval predation by birds (e.g., starlings, East and Pottinger, 1975). In order to prevent economic losses, optimal density of predators and distribution of refugia in fields must be determined (Collins et al., 2002).

Arthropod predators known to prey upon African black beetle in its native range and also in other regions include scoliid and tiphiid (Hymenoptera), tachinid flies and a number of beetles belonging to the families Carabidae, Staphylinidae, and Elateridae (Cameron et al., 1979). However, only carabids of the genus *Scarites* have been found to be significant as their populations are more abundant and stable than some of the other predators (Valentine, 1979). Even though carabids are considered potential pest-control agents because of their wide range of prey (Kromp, 1999), we currently have limited knowledge on their ecology in Australia and how efficient they are at controlling particular pest species, including African black beetle (Horne and Page, 2008).

Some of the vertebrate predators that have been reported to consume African black beetle include the Amur falcon (*Falco amurensis*), lesser kestrel (*Falco naumanni*) (Pietersen and Symes, 2010), starling (*Sturnus vulgaris*) (East and Pottinger, 1975), Hadeda ibis (*Bostrychia hagedash*), the cattle egret (*Bubulcus ibis*), Guinea fowl (Numididae), moles and rodents (Valentine, 1979). There is a lack of information on the vertebrate predators of African black beetle in Australia but from some publications the straw-necked ibis (*Thresbiornis spinicollis*), white ibis (*Threskiornis moluccus*) (Carrick, 1959), the Australian magpie (*Cracticus tibicen)* and the Australian raven (*Corvus coronoides*) (Ford et al., 2001) have been cited to consume them in highly infested areas. Consequently these bird species are often used as a cue for selecting beetle sampling sites. In addition, foxes (*Vulpes vulpes*) at times heavily rely on insect consumption, but in these particular cases it is usually related to availability of the insect species or population levels; remains of pasture beetles *Aphodius howitti* and *Rhopaea heterodactyla* have been found in stomachs of several foxes (Coman, 1973). Similarly, from field observations, fecal pellets (which are believed to be from fox) containing the exoskeleton of the beetles have been found on paddocks where African black beetles were abundant. However, the effectiveness of biological control by these predators is limited to their abundance and to the areas in which they coexist with their prey (East and Pottinger, 1975). Moreover, vertebrate predators may cause damage to pastures (i.e., scratch the top soil) in order to locate and subsequently feed on scarab larvae (Georgis et al., 2006).

## Entomopathogens

### Nematodes

Entomopathogenic nematodes (EPNs) of the families Heterorhabditidae and Steinernematidae are often used as biological control agents of economically important insect pests due to the fact they are obligate parasites (Shapiro-Ilan et al., 2012). Unlike some parasitic nematodes, EPNs have a mutualistic relationship with pathogenic bacteria of the genera *Photorhabdus* for Heterorhabditidae, and *Xenorhabdus* for Steinernematidae (Lacey et al., 2001; Lewis et al., 2006). Both genera of symbiotic bacteria are motile and gram-negative Enterobacteria (Burnell and Stock, 2000). A number of pasture pests can be managed using nematodes including white grubs (Coleoptera: Scarabaeidae), mole crickets (*Scapteriscus* spp.), billbugs (*Sphenophorus* spp.), and the black cutworm (*Agrotis ipsilon*) (Georgis et al., 2006).

The process of infection is that juvenile nematodes seek out a suitable host to attach and penetrate (Lewis et al., 1993). Host penetration can occur through thin parts of the cuticle, spiracles (tracheae), mouth, and anus (midgut) (Koppenhöfer et al., 2000). The nematode-bacterial complex becomes lethal once it reaches the haemocoel, where the bacteria are released and multiply, killing the host within 48 h (Lewis et al., 1993; Lacey et al., 2001). When seeking potential pathogenic agents, Longworth and Archibald (1975) found that a nematode, *Neoaplectana* sp. (Steinernematidae), was present in African black beetle larvae.

However, there are a number of limitations for the use of EPNs as pest control agents. Factors that affect EPNs include accumulation of thatch in soil, soil temperatures below 20°C, soil texture (fine is better), moisture retention, and irrigation (Georgis and Gaugler, 1991). At present the costs of using EPNs are much higher than those associated with use of commercially available chemical insecticides (Georgis et al., 2006), making them economically nonviable. It has been suggested that EPNs may play an important role in integrated pest management (IPM) in the future as insects become more resistant to pesticides (Lacey et al., 2001). There is evidence of synergism between imidacloprid and EPNs against third instar scarab larvae (Koppenhöfer et al., 2000). However, imidacloprid efficacy decreases against scarabs in the latter stages of larval development (third instar), which are known to cause the most damage (Lacey et al., 2001).

### Bacteria

There are a number of potential bacterial control agents for insect pests of pastures.

#### Bacillus thuringiensis

*Bacillus thuringiensis* (*Bt*) is a gram positive spore-forming bacterium that has been widely suggested as a biological control agent against agricultural pests (Kati et al., 2007). The insecticidal properties of *Bt* are associated with Cry proteins (δ-endotoxins) which are synthesized as parasporal crystals during sporulation of the bacteria (Deml et al., 1999, Figure 3). Different varieties of *Bt* produce different toxins that are specific to targets from Lepidoptera to Diptera and Coleoptera (Cidaria et al., 1991). Four major classes of insecticide crystal protein (ICP) genes have been identified: Lepidoptera-specific (CryI or Cry1), Lepidoptera- and Diptera-specific (Cryll or Cry2), Coleoptera-specific (CryIII or Cry3), and Diptera-specific (CryIV or Cry4) proteins (Chambers et al., 1991).

**Figure 3:**
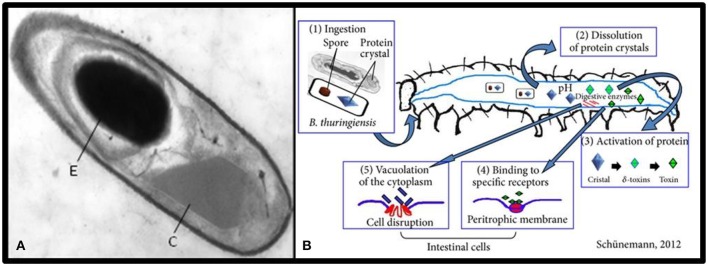
**(A)** Transmission electron micrograph of a longitudinal section of *Bacillus thuringiensis* toward the end of sporulation; the spore (E) and the crystal inclusion (C) (Sanchis, 2010). **(B)** The mode of action of *Bacillus thuringiensis* (Schünemann et al., 2014).

A study evaluating the effects of *Bt*-maize expressing Cry1Ab on non-target species found that there was no effect on mortality, mass, fertility, or fecundity of *Heteronychus arator* and *Somaticus angulatus* (Coleoptera) (Erasmus and Berg, 2014). However, there is evidence of cross-order toxicities occurring on target species, for example, Cry1Ab affecting mosquitoes *Aedes aegypti* (Diptera) (Haider et al., 1986), and CryIIIA having a comparable toxicity to CryIA in a number of caterpillars (Lepidoptera) (Deml et al., 1999). Van Frankenhuyzen (2009) highlighted that cross-order toxicities have been reported for 15 of the 87 insecticidal crystal protein families and that these numbers are likely to increase as testing across orders is expanded. Furthermore, *Bt* toxins have been found to affect mortality, development and longevity of parasitoid species that use target Lepidoptera as a host (Romeis et al., 2006).

Currently two methods are used to deliver *Bt* insecticidal proteins: formulated products prepared from naturally occurring or conjugated strains and development of transgenic plants which possess the genes responsible for the production of the toxin (Lacey et al., 2001). Some of the *Bt*-transgenic crops used include potatoes (Arpaia et al., 2000), eggplant (Arpaia et al., 2007), cotton (Sivasupramaniam et al., 2008), rice (Han et al., 2015), tobacco (Gore et al., 2005), and maize (Erasmus and Berg, 2014).

#### *Paenibacillus* sp.

Milky disease in scarab beetles receives its name from the milky aspect of the larva caused by a build-up of bacterial spores and parasporal bodies in the blood (Klein and Kaya, 1995). Milky disease comprises a number of species and strains of spore-forming rod bacteria which differ in morphology and virulence to specific hosts (Steinkraus and Tashiro, 1967). Milky disease bacteria can be found in scarab populations on all continents (Jackson and Klein, 2006). *Paenibacillus popilliae* and *Paenibacillus letinmurbos* (formerly *Bacillus*) are responsible for causing milky disease in Japanese beetle (*Popillia japónica* N.) and several other members of the scarab family (Dutky, 1940; Beard, 1956; Pettersson et al., 1999; Stahly et al., 2006). Bacterial spores are consumed by larvae while feeding on plant roots. Once the spores reach the gut, germination takes place followed by penetration of the haemocoel by vegetative cells (Harrison et al., 2000). Vegetative growing bacteria then sporulate in an asynchronous fashion leading to the death of the larvae (Rippere et al., 1998). Infectivity of *P. popilliae* varieties among scarabs tends to be higher in the species from which they were isolated (Klein and Kaya, 1995).

Dutky (1940) described the differences between the milky disease caused by *Paenibacillus popilliae* and *Paenibacillus letinmurbos*, describing them as type A and type B, respectively. Macroscopically they cannot be distinguished, however, the general appearance is quite different. In type A larvae tend to have a milk white coloration while in type B larvae turn muddy brown color. This coloration is due to the formation of haemolymph clots which block the insects' circulation resulting in gangrenous condition of the affected parts (Stahly et al., 2006).

Similar to *Bt, P. popilliae* produces parasporal crystals upon sporulation (Klein and Kaya, 1995; Deml et al., 1999). It has been suggested that parasporal crystal proteins may play a part in the mortality caused by milky disease because of the strong similarities and conservation of the hydrophobicity distribution of Cry proteins from *Bt* and *P. popilliae* (Zhang et al., 1997). While most of the insecticidal activity of *Bt* has been linked with the proteinaceous toxins located in parasporal inclusion bodies (parasporal crystals) (Lacey et al., 2001), *P. lentimorbus* causes a disease that is almost identical to that caused by *P. popilliae*, and has no parasporal inclusion (Stahly et al., 2006).

Despite the limitations of infectivity due to specificity from different varieties of *P. popilliae* (Klein and Kaya, 1995) this bacterium relies on the presence of viable spores to infect its host as vegetative cells experience a decrease of viability in soil as well as deficient virulence (Stahly and Klein, 1992). Similarly with *Bt*, Head et al. (2002) demonstrated that Cry proteins accumulated in soil due to the continuous use of transgenic *Bt* cotton are subsequently incorporated into the soil resulting in no detectable immunological and biological activity. Furthermore, other factors such as application of insecticides and fungicides have been shown to affect spore viability in soil (Dingman, 1994).

#### *Serratia* sp.

Bacteria which cause Amber disease have been mentioned in the literature as a potential biological control option for scarabs (Jackson and Klein, 2006). *Serratia entomophila* (Enterobacteriaceae) is a gram-negative nonspore-forming, non-encapsulated, straight rod bacterium with peritrichous flagellae (Grimont et al., 1988). Pathogenic strains of *S. entomophila* infect their host by colonizing the larval gut and adhering to the crop; as a result starvation is induced causing the depletion of the fat bodies (Klein and Kaya, 1995). Consequently, this series of events ultimately causes the appearance of an amber color (Jackson et al., 1993), which gives its name to the disease (Klein and Kaya, 1995). It has been found that pathogenic strains of *S. entomophila* and *S. proteamaculans* causing amber disease contain a specific plasmid (Hurst et al., 2000). However, despite intensive testing, no other scarab species apart from *Costelytra zealandica* has been found susceptible to the plasmid-bearing strains (Jackson and Klein, 2006).

#### *Rickettsiella* sp.

*Rickettsiella* sp. is another bacterium that has been isolated from African black beetle and thus highlighted as a potential biological control agent for scarabs. Along with a protozoan, possibly *Adelina* sp., *Rickettsia* sp. was the most abundant pathogen isolated by Longworth and Archibald (1975). The genus *Rickettsiella* is made-up of intracellular bacterial pathogens of a wide range of arthropods (Leclerque et al., 2011). They are characterized by causing intracoelomic infections, multiplying in vacuolar structures within fat body cells and are often associated with protein crystals (Kleespies et al., 2011). However, infected larvae may live for several months (Longworth and Archibald, 1975).

## Protozoa

Protozoan control agents offer persistence in host populations while decreasing overall fitness and reproduction of the target species, however, they produce low levels of immediate mortality (e.g., chronic infections) and *in vivo* production is required to prepare and release overwhelming amounts of the control agent (inundative application) (Lacey et al., 2001).

## Virus

Isolations from diseased larvae and adults of African black beetle have revealed a number of pathogens, including a small isometric virus (30 nm in diameter) that develops in the cytoplasm of gut and fat-body cells (Longworth and Archibald, 1975). Longworth and Carey (1976) described this RNA virus as being icosahedral in shape without any obvious surface features. Moreover, it has a sedimentation coefficient of 137S, a buoyant density in CsC1 of 1.33 g/ml and RNA:protein ratio of 28.2:71.8. The virus was found to be infective for numerous species in the Lepidoptera and Coleoptera orders and also for *Drosophila melanogaster* cells in tissue culture (Crump and Moore, 1981). However, the low infection rate of the virus on African black beetle observed by Longworth and Archibald (1975) in the field was not enough to explain the mortality observed in the population.

## Fungi

Entomopathogenic fungi of the genera *Metarhizium* and *Beauveria* are omnipresent in soils; however, infectivity in scarabs is limited to certain strains mostly of the species *B. brongniarti* and the large-spored *M. anisopliae* var. *majus* (Jackson and Klein, 2006). *Beauveria* sp. has been isolated from African black beetle larvae (Longworth and Archibald, 1975) and has been considered as a potential biological control agent. Zimmermann (2007) summarized the infection pathway of *Beauveria* sp. and other entomopathogenic fungi in a sequence of events: attachment of the spore to the cuticle, germination, penetration of the cuticle, overcoming the immune response of the host, proliferation, saprophytic outgrow from the carcass and production of new conidia. However, when considering entomopathogenic fungi as biological control agents for soil-dwelling species, the ability of the entomopathogen to persist for an extended period of time as well as its infectivity to the host must be taken into account (Lingg and Donaldson, 1981). The survival and proliferation of these fungi can be affected by a number of abiotic factors such as temperature, humidity or moisture and solar radiation (Zimmermann, 2007).

## Chemical control

Management of subterranean pest species such as African black beetle is challenging because of the high damage potential per individual, therefore the success of any control method(s) depends on the reduction of the population to the minimum (Bulinski and Matthiessen, 2002). Many of the pesticides previously used for African black beetle control have either been withdrawn from the market or are no longer registered for that purpose. Traditionally, persistent broad-spectrum organochlorine products were deployed with cultivation and incorporated into the soil in order to protect crops from African black beetle (Bulinski and Matthiessen, 2002). In recent years, targeted insecticides such as insect growth regulators and neonicotinoid compounds have been developed (Jackson and Klein, 2006). Imidacloprid (Merit, Bayer, Kansas City, MO, USA) and halofenozide (Mach 2, RohMid, Parsippany, NJ, USA) have become widely used for preventive control of root-feeding scarabaeid grubs (Kunkel et al., 2001). However, Kunkel et al. (1999) found that imidacloprid and halofenozide may have disruptive effects on earthworms and some predatory invertebrates, but such effects are short-lived and unlikely to cause pest outbreaks. In contrast, Prabhaker et al. (2011) found limited but detrimental effects of neonicotinoid compounds (imidacloprid and thiamethoxam) on some beneficial insects and maintained a more conservative approach, arguing that further investigation is required.

Imidacloprid and halofenozide are most effective against early larval instars (first and second instars), and must be applied before larval damage is visible (Jackson and Klein, 2006). However, third instar larvae are known to cause the most damage (Lacey et al., 2001). When evaluating spring and autumn applications of chlorpyrifos, alpha-cypermethrin, and diazinon for African black beetle control, Eden et al. (2011) concluded that the use of such pesticides is not recommended because of the difficulty in application timing, the inefficiency of treatments, and the likelihood that reinvasion will occur as these treatments do not prevent subsequent larval populations from causing damage.

## Seed treatment

It has been suggested that treated seeds (i.e., dressing, film coating, pelleting, or multilayer coating) present an environmentally safe method of protection for young plants against insect pests (Elbert et al., 2008). Seeds coated with insecticides (imidacloprid and furathiocarb) produce plants that are protected against stem borers (e.g., African black beetle) through systemic translocation of the insecticides (Drinkwater and Groenewald, 1994). However, Drinkwater (2003) found that in order to deter beetles, they have to feed on the plant first. Furthermore, a number of biological factors such as the age of the beetle influence the level of efficacy of the compound (Drinkwater, 2002). Drinkwater (2003) concluded all neonicotinoids evaluated significantly reduced insect damage to the host plant, but only imidacloprid reduced beetle abundance. Bell et al. (2011) suggested that treated seeds might play a crucial role in pasture establishment during outbreak years, as well as helping to control population numbers and avoid the risks of population build-up after pasture renewal.

## Silicon supplementation

Plant silicon is known to play a role in defense against pathogens and herbivores (Epstein, 2009). In grasses, silicon-based defenses provide a physical barrier that counters herbivores and pathogens (Massey et al., 2006; Massey and Hartley, 2009; Reynolds et al., 2009).

Massey and Hartley (2009) demonstrated that silica-rich diets increase mandible wear and decrease digestibility and absorption of nitrogen from food plants in African armyworm (*Spodoptera exempta*). In addition, silicon can also affect subterranean herbivores. Frew et al. (2016) found that silicon applications can play a significant role in defense against root-feeding pests such as greyback cane beetle larvae (*Dermolepida albohirtum*).

Silicon is the second most abundant element in soils (Epstein, 1994) but needs to be in the soluble form of monosilicic acid [Si(OH)_4_] to be taken up by the plant roots (Guével et al., 2007). Once metabolized, silicon can provide a physical defense based on the mechanical properties of opaline silica (Garbuzov et al., 2011). Silicon concentrations within a grass species are not static but can increase when the plant is under herbivore attack (Massey et al., 2007), suggesting that there is a fitness cost associated with this defense (Garbuzov et al., 2011). It is thought that silicon defense fitness costs might place the plant at a disadvantage against its competitors in the absence of herbivores (Hanley and Sykes, 2009).

Silicon supplementation has shown promising results at deterring herbivores above and below ground (Massey and Hartley, 2009; Frew et al., 2016). It relies, however, on the availability of soluble silicon (Guével et al., 2007) and herbivore stimuli for plants to invest in this defense strategy (Massey et al., 2007). Silicon supplementation could complement other management strategies such as chemical defenses that can improve overall plant health and resistance (i.e., endophytes). However, it is important to take into account that silicon may reduce digestibility and grazing preference in vertebrates (e.g., sheep, Glenn et al., 1989).

## Endophyte as a control method

*Epichloë* (syn. *Neotyphodium*) has been described in early publications as an endophytic fungus of grasses such as perennial ryegrass and tall fescue (Sampson, 1933). Although endophytes are inconspicuous *in planta* (Iannone et al., 2011), infected plants can experience increased growth, reproduction, and resistance to various biotic and abiotic stress factors (Clay and Schardl, 2002). Biotic resistance of endophyte-infected plants has been associated with an array of secondary metabolites (alkaloids) produced by the fungus that benefit the host plant as they provide resistance against herbivores and pathogens (Siegel et al., 1987; Wilson, 1993; Zain, 2011). Toxicosis in cattle and sheep has been associated with the ingestion of endophyte-infected pastures, decreasing animal performance, and in some cases causing death (Fletcher and Harvey, 1981; D'Mello and MacDonald, 1997). It has been determined that ergopeptine (ergovaline) alkaloids are responsible for causing tall fescue staggers or fescue toxicosis (Paterson et al., 1995), while isoprenoid lolitrem (lolitrem B) alkaloids are responsible for causing ryegrass staggers (Smith et al., 1997).

On the other hand, some alkaloids have proven to be beneficial, conferring insecticidal properties to the plant, such as the pyrrolopyrazine alkaloid peramine that acts as a feeding deterrent to the Argentine stem weevil (Rowan and Gaynor, 1986; Rowan, 1993) and epoxy-janthitrems (indole-diterpenes) which are produced by an endophyte variety called AR37 (Thom et al., 2014). Epoxy-janthitrems have been reported not to cause ryegrass staggers in cattle (Moate et al., 2012). In addition, loline has shown both feeding deterrence and insecticidal activity (Schardl et al., 2007), while only causing negative effects in mammals at extremely high concentrations (Strickland et al., 1994; Oliver et al., 1998).

When comparing livestock performance on endophyte-infected and endophyte-free swards, Prestidge et al. (1982) found that non-infected pasture was severely damaged by the Argentine stem weevil, highlighting the importance of the endophyte (Prestidge et al., 1982). Endophytes have been reported to offer protection against a number of insect pests, including black cutworm (*Agrotis ipsilon*) (Baldauf et al., 2011), pasture mealybug (*Balanococcus poae*) (Pennell et al., 2005), Argentine stem weevil (Prestidge and Gallagher, 1988b), root aphids (Popay and Thom, 2009), and Japanese beetle and other white grubs (Scarabaeidae spp.) (Potter et al., 1992) including African black beetle (Bell et al., 2011).

In a field trial comparing different grass treatments, African black beetle populations in perennial ryegrass pastures harboring AR37, AR1, and wild-type endophyte remained low and their mean densities in these treatments were significantly less than those pastures without endophyte (Thom et al., 2014). Therefore, considerable research has been done on the alkaloids produced by fungi because of the services they provide to their host plant and agricultural systems (Clay and Schardl, 2002).

It has been found that the level of chemicals produced by an endophyte in symbiosis with a plant varies depending on the endophyte-plant combination (Panka et al., 2013). This is particularly important as endophyte-infected grasses containing ergot alkaloids (ergovaline) that are known to have detrimental effects on cattle (Smith et al., 1997; Bell et al., 2011) can also deter important pests such as African black beetle (Ball et al., 1997b). Therefore, screening endophyte-plant combinations to find a balanced chemical profile that protects the plants from pests without affecting cattle would be beneficial. In order to achieve this, it is necessary to understand the chemistry behind these processes (i.e., active compounds, intermediate compounds, and possible synergistic effects).

Ball et al. (1997b) tested alkaloid toxicity on adult African black beetle by incorporating them in an artificial diet, and found that ergopeptine alkaloids significantly reduced feeding at concentration of 5 μg/g, whereas ergopeptine epimer and its analogs were also active but to a lesser extent. In addition, he found that peramine, lolitrem B and a number of ergot alkaloids had no effect on deterring adult beetles, except for ergonovine which showed moderate activity.

As for insecticides (Jackson and Klein, 2006), the effects of endophyte infected grasses on African black beetle can vary depending on their different life stage. Previous studies have demonstrated that certain endophyte strains deter adult beetles from feeding (Ball et al., 1994), resulting in a decrease of survival and oviposition. However, commercially available endophyte strains do not seem to have negative effects on the larval form (Bell et al., 2011). Similarly, Watson (2006) found no evidence of alkaloids produced by endophyte-infected perennial ryegrass or tall fescue affecting redheaded cockchafer and black headed cockchafer larval stages. However, Bryant et al. (2010) found loline concentrations in excess of 1700 μg/g DM were particularly effective in reducing feeding and development of second instar redheaded cockchafer but not African black beetle larvae.

Endophytes have been screened to produce less toxic profiles to livestock, whilst maintaining other beneficial traits, such as the production of insect deterrent alkaloids (Johnson et al., 2013). In the case of vertebrates, it has been determined that some toxic alkaloids, such as ergot alkaloids, behave like neurotransmitters (i.e., dopamine, serotonin, and adrenaline) causing vasoconstriction, smooth muscle contraction, bewilderment, and hallucinations (Beaulieu et al., 2013). In addition, penitrem, paxilline, and lolitrem B are also known to be tremorgenic and have been associated with diseases of domestic animals and mice (Gallagher et al., 1981; Smith et al., 1997). However, the exact mechanisms of action of how endophytes affect soil-borne herbivores are still to be determined (Malinowski and Belesky, 2000). In a bioassay conducted on Argentine stem weevil, Rowan (1993) found definite but minimal structural requirements for insect deterrence activity caused by peramine and its analogs. All analogs tested were less active than peramine itself, suggesting some importance for the guanidinium group and the side-chain in obtaining the full biological response. A better understanding of the mode of action of alkaloids on soil-borne insects might provide valuable information for the development of novel endophytes to control the more resistant life-stage (i.e., larval form).

Clay (1989) suggested that an efficient biological control agent is characterized by its capacity to significantly decrease pest damage either by directly killing or damaging the pest, reducing its population growth, or by deterring the pest before it can do any damage. Endophytes have shown to offer insect deterrent activity to their host plant against certain pests as well as inducing resistance in their host plant to various other biotic factors (Clay and Schardl, 2002).

Even though there are a number of pathogens associated with scarabs, it appears that many occur at low levels and scarabs appear to show inherent resistance to many generalist pathogens (Jackson and Klein, 2006). In contrast, endophytes as a control method have a clear advantage, as they are present within host-plant grasses (Iannone et al., 2011) and they are transmitted vertically through seeds (Schardl, 1996). Therefore, in terms of presence, endophytes can be expressed in paddocks offering continued protection to their host plant. In addition, the chemical profile produced by an endophyte, in symbiosis with a plant, varies depending on the endophyte-plant combination (Panka et al., 2013) and as a result, endophyte-plant combinations could be selected according their chemical profile and the target pest affecting the plant.

Wild-type endophyte-infected grasses which contain lolitrem B and ergovaline offer insect control, but consumption by dairy cows may result in ryegrass staggers, reduction in feed intake, and losses in milk production (Thom et al., 2014). In recent years, development efforts have focused upon endophyte—grass host associations that produce little or no ergot alkaloids toxic to livestock yet still maintain pest resistance qualities of the more toxic profiles (Malinowski and Belesky, 2000).

Bell et al. (2011), however, states that endophytes could face limitations during African black beetle outbreaks, as insect-deterrence conferred by the best selected endophytes may not be sufficient to prevent larvae population build up or new infestations arising as a result of flight dispersal by adult beetles in late autumn or spring. Furthermore, Jackson and Klein (2006) concluded that while chemical control will be still used as a quick fix for scarab problems, integrated pest management (IPM) offers a better long-term solution. Integrated pest management (IPM) refers to the synergistic use of multiple control strategies (e.g., cultural, chemical, and biological) based on surveillance information to assess and control pests in an ecologically and economically sound manner (All, 2005; Ehler, 2006). Kauppinen et al. (2016) proposed that *Epichloë* endophytes should be considered when developing sustainable management strategies for agriculture, as endophyte-infected grasses could be used as alternatives and/or in conjunction with synthetic plant protection products.

In view of the above, the use of endophytes may aid to control insect pest populations and therefore reduce the need for pesticide applications in the field. Prabhaker et al. (2011) argues that even though neonicotinoid compounds (imidacloprid and thiamethoxam) used for soil-borne insects are generally assumed to be safe they can have negative effects on beneficial insects (e.g., via food chain toxicity; following feeding on plant tissue or excretions) if they are exposed to the pesticide.

Consequently, the use of endophytes might allow the recruitment of natural predators which have been suggested to play a key role in the prevention of pest outbreaks in sustainable agricultural systems (Kromp, 1999). Riechert (1999) argues that the presence of natural predators can affect pest species by deterring pests, causing them to utilize unsuitable areas that are less productive, or even cease feeding completely; ultimately, delaying the outbreak phase by controlling population numbers when they are below plague levels.

Endophytes may play a crucial role in IPM in sustainable agricultural systems, as they not only enhance host plant resistance to biotic factors but also to abiotic factors (Clay and Schardl, 2002). Some examples of IPM approaches for scarab control include the combination of entomopathogenic nematodes *Heterorhabditis megidis* and *Steinernema glaseri* with *Metarhizium anisopliae* (Ansari et al., 2004) and also the combination of imidacloprid with entomopathogenic nematodes (Koppenhöfer et al., 2000). However, the combination of two different control strategies does not necessarily result in the desired outcome. Walston et al. (2001) found a lack of synergism between endophyte infected perennial ryegrass and *P. popilliae* on Japanese Beetle. Perhaps a combination of chemical agents, such as targeted pesticides (e.g., imidacloprid) applied as seed treatments plus the application of soluble silica could help establish endophyte-infected grass when renewing pastures, and supplement entomopathogenic nematodes and other natural predators in an IPM strategy.

To conclude this literature review it is apparent that African black beetle is not a pest that can be controlled with a single strategy and requires a more holistic approach. The design of an IPM program that works on-farm is necessary. To achieve this, different methods of control that are synergistic should be aligned with farmers needs and capabilities. Based on the advantages described in this review, selection of an ideal endophyte-grass combination could be a first step to develop such an IPM program.

## Author contributions

MK: researched the relevant literature and wrote the body of the article. AY: contributed with information regarding insects and ecology, as well as, editing the article. SR: contributed mainly in the biochemistry part of the article, information about secondary metabolites, as well as, editing the article. KG: contributed with information about ryegrass endophyte, as well as, editing the article. KP: edited the final version of the article. JE: edited the final version of the article. GS: provided overall direction for the program of work.

### Conflict of interest statement

The authors declare that the research was conducted in the absence of any commercial or financial relationships that could be construed as a potential conflict of interest.
